# Characterization of Seizure Induction Methods in *Drosophila*

**DOI:** 10.1523/ENEURO.0079-21.2021

**Published:** 2021-08-20

**Authors:** Jurga Mituzaite, Rasmus Petersen, Adam Claridge-Chang, Richard A. Baines

**Affiliations:** 1 Division of Neuroscience and Experimental Psychology, School of Biological Sciences, Faculty of Biology, Medicine and Health, University of Manchester, Manchester Academic Health Science Centre, Manchester M13 9PL, United Kingdom; 2 Institute for Molecular and Cell Biology, A*STAR, Singapore 138673; 3 Department of Physiology, National University of Singapore, Singapore 117593; 4 Program in Neuroscience and Behavioral Disorders, Duke-NUS Medical School, Singapore 169857

**Keywords:** antiepileptic, *Drosophila*, epilepsy, insect, model, seizure induction

## Abstract

Epilepsy is one of the most common neurologic disorders. Around one third of patients do not respond to current medications. This lack of treatment indicates a need for better understanding of the underlying mechanisms and, importantly, the identification of novel targets for drug manipulation. The fruit fly *Drosophila melanogaster* has a fast reproduction time, powerful genetics, and facilitates large sample sizes, making it a strong model of seizure mechanisms. To better understand behavioral and physiological phenotypes across major fly seizure genotypes we systematically measured seizure severity and secondary behavioral phenotypes at both the larval and adult stage. Comparison of several seizure-induction methods; specifically electrical, mechanical and heat induction, show that larval electroshock is the most effective at inducing seizures across a wide range of seizure-prone mutants tested. Locomotion in adults and larvae was found to be non-predictive of seizure susceptibility. Recording activity in identified larval motor neurons revealed variations in action potential (AP) patterns, across different genotypes, but these patterns did not correlate with seizure susceptibility. To conclude, while there is wide variation in mechanical induction, heat induction, and secondary phenotypes, electroshock is the most consistent method of seizure induction across known major seizure genotypes in *Drosophila*.

## Significance Statement

Epilepsy is a neurologic disorder affecting one in 130 people globally, with a significant impact on patients, families, and society. Approximately one third of epileptics do not respond to currently available medication. Thus, better insights into underlying disease mechanisms and identification of new drugs are needed. Fruit flies (*Drosophila melanogaster*) are a powerful genetic model: a number of single gene mutant flies exhibit seizures, phenotypes that have been shown to respond to established antiepileptic drugs (AEDs). We compare methods of seizure induction and their utility, to establish which induction method is the most consistent across a range of different seizure-inducing genetic backgrounds. Adopting a common method for seizure analysis in this model will, we predict, speed identification of novel anti-convulsive treatments.

## Introduction

Epilepsy is one of the most common neurologic disorders, affecting ∼60 million people worldwide (Chen et al., 2018). While a variety of causes contribute to epilepsy, including traumatic brain injury and brain infections, the major contribution is from underlying genetic mutations (Poduri and Lowenstein, 2011). It has been estimated that ∼70% of epilepsies do not have a single known cause; of these, 60% have been associated with genetic mutations (Heron et al., 2007; Poduri and Lowenstein, 2011). There are around 700 identified gene mutations currently associated with epilepsy. These include genes contributing to planar cell polarity and the noncanonical WNT signaling pathway (e.g., PRICKLE1), autism spectrum associated genes (e.g., AUTS2), and mTOR signaling pathway genes (e.g., mTOR and TSC1; Bassuk et al., 2008; Citraro et al., 2016; Wang et al., 2017). However, a majority of epilepsy genes directly influence ion-channel function, specifically mutations in voltage-gated sodium, potassium, and calcium channels.

Clinicians have access to over 25 antiepileptic drugs (AEDs) to minimize epileptic seizures (Löscher and Schmidt, 2011). A majority of these drugs target ion channels or neurotransmitter signaling, in an attempt to re-establish an appropriate balance between excitatory and inhibitory signaling in the brain (Löscher and Schmidt, 2011; Vezzani et al., 2011). Although many new AEDs have been approved in recent years, many new drugs have proven no more effective in treating drug-resistant epilepsies than older compounds (Schmidt and Schachter, 2014; Moshé et al., 2015; Chen et al., 2018). It seems likely that treating drug-refractory epilepsy will require novel drug targets and therefore a deeper understanding of epilepsy at the level of basic mechanism(s).

**Figure 1.**
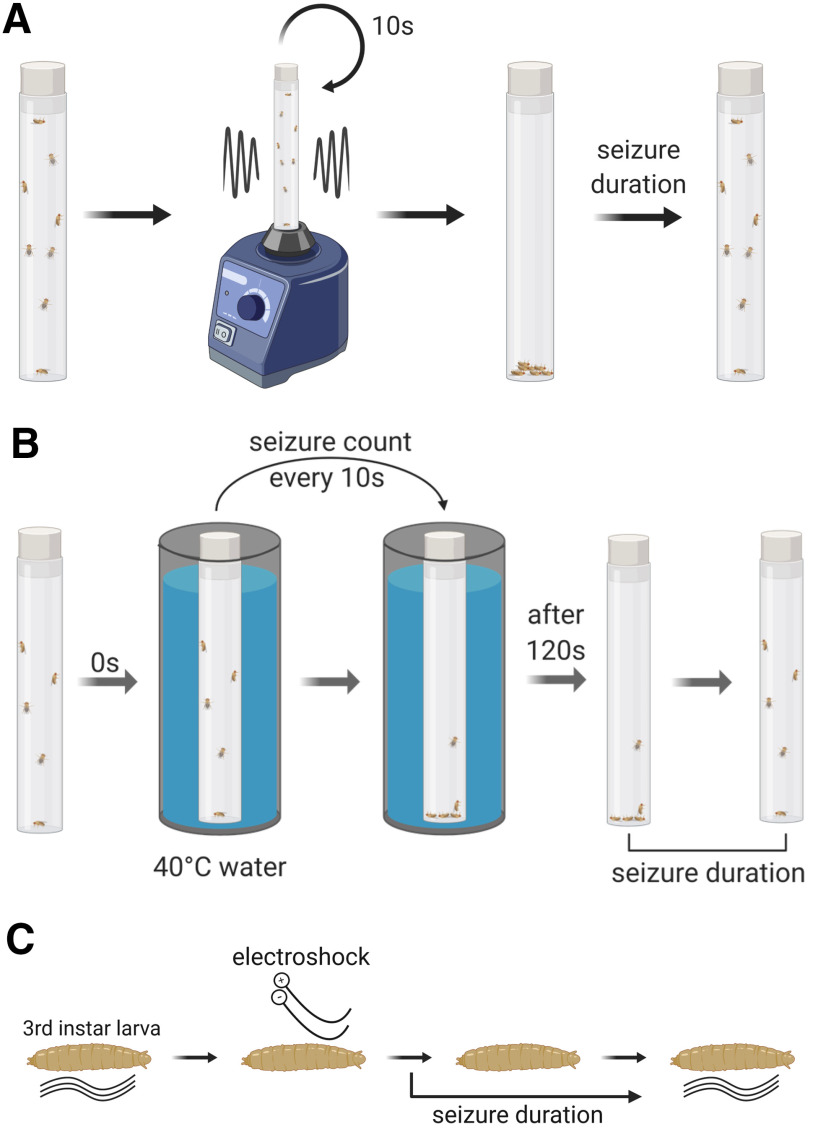
Methods of seizure induction in *Drosophila* adults and larvae. ***A***, Schematic showing vortex assay. Adult flies in vials are vortexed for 10 s, and their seizure duration is measured as time taken to regain their posture. ***B***, Schematic showing heat assay. Adult flies in vials are exposed to 40°C water for 120 s, and seizure duration is measured as time taken to regain their posture after being taken out from the water bath. ***C***, Schematic showing electroshock assay. Electroshock is applied to the dorsal side of third instar larvae. Seizure duration is measured as the time required to restart normal crawling after the shock.

In the genetic model *Drosophila*, both wild-type and mutant animals exhibit seizure-like behaviors; mutants undergo seizures with greatly reduced stimulus thresholds and/or seizure-like activity (SLA) lasts far longer (Ganetzky and Wu, 1982; Pavlidis et al., 1994; Kuebler and Tanouye, 2000; Parker et al., 2011a; Baines et al., 2017). In the adult fly, SLA includes repetitive proboscis extension, wing buzzing and loss of posture (Tan et al., 2004; Parker et al., 2011a). These SLAs have formed the basis of a variety of seizure-severity assays (Pavlidis and Tanouye, 1995). Several reviews have previously characterized fly electrophysiological and behavioral responses to specific seizure assays (Pavlidis and Tanouye, 1995; Kuebler and Tanouye, 2000; Parker et al., 2011a; Lee et al., 2019). In adult flies, seizure-susceptible mutants fall into two main categories based on seizure induction: mechanical (termed bang sensitive; BS) and temperature induced (Ganetzky and Wu, 1982; Kasbekar et al., 1987; Pavlidis and Tanouye, 1995; Burg and Wu, 2012). Mechanical induction has been standardized with the use of a laboratory vortexer to hyperstimulate sensory inputs and is termed the “vortex assay” (Kuebler and Tanouye, 2000; Fig. 1*A*). By contrast, the heat assay exploits temperature change to induce seizure (Burg and Wu, 2012; Saras and Tanouye, 2016; Fig. 1*B*). There is a third, and more involved seizure induction method in adult flies, known as high-frequency stimulation of the giant fiber (GF) pathway (Pavlidis and Tanouye, 1995). This method induces seizures in all BS and other seizure genotypes like *shaker* (Lee and Wu, 2006). While this assay allows investigation of synaptic transmission during seizures, stimulation of the GF pathway requires a more complex set up than either the vortex or temperature-shock assays and is not suitable for medium-throughput or high-throughput screening.

In larvae, seizures have been induced using a simplified electroshock assay, during which the whole body is subjected to electroshock (Marley and Baines, 2011; Fig. 1*C*). A particular advantage of larvae is that they are well-suited to drug screening and, moreover, provide unparalleled understanding of CNS structure and function (Choi et al., 2004; Worrell and Levine, 2008; Marley and Baines, 2011; Kadas et al., 2015; Lemon et al., 2015).

Genetic epilepsies include syndromes characterized by febrile (heat-“fever”-induced) seizures that often present in children. Genetic epilepsy with febrile seizures plus (GEFS+) is commonly caused by sodium channel mutations (Camfield and Camfield, 2015). Some extreme cases of GEFS+ are classified as Dravet syndrome (DS). This often affects children in their first year of life, and has additional comorbidities including motor and mental impairments (Ziobro et al., 2018). DS is typically resistant to AED treatment necessitating additional research. Use of simpler model organisms, including *Drosophila*, are well suited to investigate mechanisms of epilepsy, but also to identify novel treatments for drug-refractory epilepsies (Schutte et al., 2014; Griffin et al., 2017).

We aimed to establish which accessible seizure-induction methods are applicable across the known range of *Drosophila* seizure mutants. We found that electroshock applied to larvae is the most reliable method to induce seizures regardless of mutation. Since epilepsy is associated with many secondary phenotypes, we investigated whether locomotor activity is altered in a predictable fashion, which could be further explored to use for drug screens. We found that there was no significant trend in the daily adult activity levels across the range of mutants tested, but mechanically-sensitive mutant larvae showed reduced crawling. Since electroshock was the most widely applicable method of seizure induction, we investigated potential common underlying mechanisms predisposing larvae to seizures. We detected elongated bursts of motor neuron firing in all seizure mutants. However, the alteration was not exclusive to seizure mutants and did not have good predictive power for seizure severity measured as seizure duration.

## Materials and Methods

### Fly stocks

Flies were maintained on standard cornmeal medium at 25°C and a 12/12 h light/dark cycle. Fly stocks were obtained from the following sources: DS (S1231R), DS-C (S1231S), GEFS+ (K1270T), and GEFS-C (K1270K) from Diane O’Dowd (Sun et al., 2012; Schutte et al., 2014); w^-^,eas^2F^;+;+, para^bss1^;+;+, Canton-S and Oregon-R from Baines lab; w^-^,+;jus^iso7.8^ from M. Tanouye; pk-sple (#422) from the Bloomington Drosophila Stock Centre.

### Adult activity assay

Male flies, aged 4–10 d posteclosion, were placed in 65-mm glass tubes containing food in one end and a foam plug at the other. The experiments were conducted at 12/12 h light/dark cycle in an environment-controlled incubator at 25°C. Fly locomotor activity was monitored for 4 d, always excluding the first 24 h from data analysis. Animal activity was measured by distance moved per hour (mm/h) and obtained using custom-build image-acquisition software (CRITTA), written in LabView (National Instruments; Mohammad et al., 2016). All tracking experiments were repeated at least twice, fly *N* numbers are listed in the respective figures. Before tracking, flies were reared at 25°C, collected on the day of eclosion and kept in vials containing only males until the day of experiment.

### Adult vortex assay

Adult flies were collected using CO_2_ 3–4 d posteclosion into empty plastic vials. Ten flies per vial were left to recover from anesthesia for 1–2 h. To assess seizure, the vials were placed on a standard laboratory vortexer at maximum speed for 10 s. Seizure duration was measured as time required to regain posture and mobility for each fly.

### Adult heat-shock assay

To measure seizure threshold and duration, adult flies were subjected to a heat assay. Male flies, aged 8–10 d posteclosion, were anesthetized using CO_2_ and placed into empty plastic fly vials, 5 flies per vial. Flies were given 1–2 h to recover. A water bath was heated to 40–41°C and kept constant at that temperature throughout the experiment. Vials containing flies were placed into the water bath and kept there for 120 s. Throughout the 2 min in the water bath, visual inspection occurred every 10 s and seizure-status noted. Seizures were identified by loss of posture and random wing buzzing. Vials were taken out of the bath after 120 s and the duration of seizure activity outside the water bath was also recorded (i.e., recovery period).

### Larval locomotion tracking

Larval locomotion was tracked using the DanioVision Observation Chamber connected to a computer with EthoVision XT software (Noldus Information Technology); 2% agarose was poured into the lid of 96-well plate and four separate arenas were cut out. Grooves between the arenas were filled with 5 m NaCl to prevent larvae from crawling off their respective stages. Individual larvae were placed on the arenas and after a 30-s adaptation period their locomotion was tracked for 3 min. Total distance crawled in the tracking period was calculated by the EthoVision XT software using centroid tracking.

### Larval electroshock assay

An electroshock assay induces seizure in third instar larvae. Wandering stage larvae were transferred from a vial containing food onto a plastic dish containing water to wash away food residue. Larvae were then transferred to an empty plastic dish and excess water gently removed with a paper towel. No more than four larvae were placed in a single dish. Once normal crawling behavior resumed a probe, composed of two conductive tungsten wires (0.1 mm in diameter, ∼1–2 mm apart), was placed on the anterior dorsal surface of a larva, over the approximate location of the CNS. A 7V DC current was applied for 2 s, generated by a DS2A Isolated Voltage Stimulator (Digimeter Ltd). In response to the shock, larvae exhibit sustained contractions of body wall muscles and occasional spasms, halting normal crawling behavior. Seizure duration was measured as a time period between the stimulus onset and resumption of normal crawling behavior. After seizure, the resumption of normal behavior involved a full peristaltic wave from either end of the animal which resulted in either forward or backward movement.

### Larval drug treatment

Gravid females and males were kept on grape agar plates (Dutscher) at 25°C. The flies were fed live yeast paste supplemented by 25 mg/ml picrotoxin (PTX; Sigma-Aldrich) stock to a final concentration of 0.125 mg/ml for 3 d before embryo collection commenced. Exposed embryos were transferred onto standard food at the end of each collection day and allowed to develop normally.

### Larval electrophysiology dissection

To record from identified aCC or RP2 motor neurons in third instar larvae, the CNS was dissected from a larva and placed in a droplet of external saline solution on a coverslip coated with a thin layer (1–2 mm) of cured SYLGARD Elastomer (Dow-Corning). The external saline solution was composed of 135 mm NaCl, 5 mm KCl, 4 mm MgCl_2_·6H_2_O, 2 mm CaCl_2_·2H_2_O, 5 mm N-Tris [hydroxymethyl]methyl-2-aminoethanesulfonic acid (TES), and 36 mm sucrose at pH 7.15. The CNS was stabilized on the coverslip using tissue adhesive (Gluture, World Precision Instruments). The aCC or RP2 cells, located in the dorsal ventral nerve cord (VNC), were exposed for recordings by removing the overlaying glial sheath using a glass pipette (GC100TF-10, Harvard Apparatus) filled with 1% (w/v) bacterial protease type XIV (Sigma-Aldrich).

### Loose patch recordings

Loose patch recordings were performed using borosilicate glass electrodes (GC100TF-10; Harvard Apparatus) pulled to resistance between 1.0 and 1.5 MΩ. The electrodes were filled with external saline of the same composition as listed above. The aCC or RP2 neurons were identified based on location in the CNS, relative soma size and axon configuration (aCC has two axons originating from the soma, RP2 has just one). An electrode was placed on a cell soma without breaking into it. Slight negative pressure was applied to suck around 1/3 of the cell into the electrode. Recordings were conducted using Axopatch 200B amplifier controlled by pCLAMP (version 10.3) using a Digidata 1322A analog-to-digital converter (Molecular Devices, Molecular Devices) or MultiClamp 700B amplifier controlled by WinWCP (version 5.1) using a Digidata 1440A analog-to-digital converter (Molecular Devices, Molecular Devices). Data were sampled at 20 kHz and low pass filtered at 0.1 kHz. All recordings were made at room temperature (18–22°C).

### Electrophysiology trace analysis

Analysis of loose patch traces was performed using a custom script written in MATLAB. Spike times were gathered using Clampfit (version 11.1). A burst is an event with a minimum of three spikes occurring within 100 ms from one another. A burst ends when no spike is detected within 100 ms following the last spike. The script provides summary data on mean burst duration and standard deviation, burst frequency, spike count per burst and firing frequency. Data were plotted using a custom Python script.

### Code accessibility

MATLAB script described in the paper is freely available online at https://github.com/jm933/BurstAnalysis.

### Statistical analysis

Data were analyzed with estimation methods using a Python custom script using the DABEST package (Ho et al., 2019). The effect size distributions shown are of the 5000 mean differences calculated in the bootstrap resampling procedure. To compare effect sizes across different assays a universal effect size measure Cohen’s *d* was used. Cohen’s *d* is an effect size where the difference has been standardized to the standard deviations of the two groups being compared (Cumming and Calin-Jageman, 2016). No significance testing was performed, *p* values were reported for legacy purposes only. Data are presented following best practices, showing observed values and effect sizes; means are provided with a standard deviation, effect-size errors are displayed as curves with 95% confidence intervals (CIs; generated with bootstrapping; Bernard, 2021).

Inferred linear relationships between various parameters were assessed using linear regression. The line was fitted and *R*^2^ was calculated using scipy library in Python using linregress function, which finds the line with the least-squares regression. Each point represents the effect size of a genotype.

Sample sizes (*N*) are the total number of animals for behavioral experiments or total number of cells for electrophysiology.

## Results

### Mechanical seizure induction

**Figure 2.**
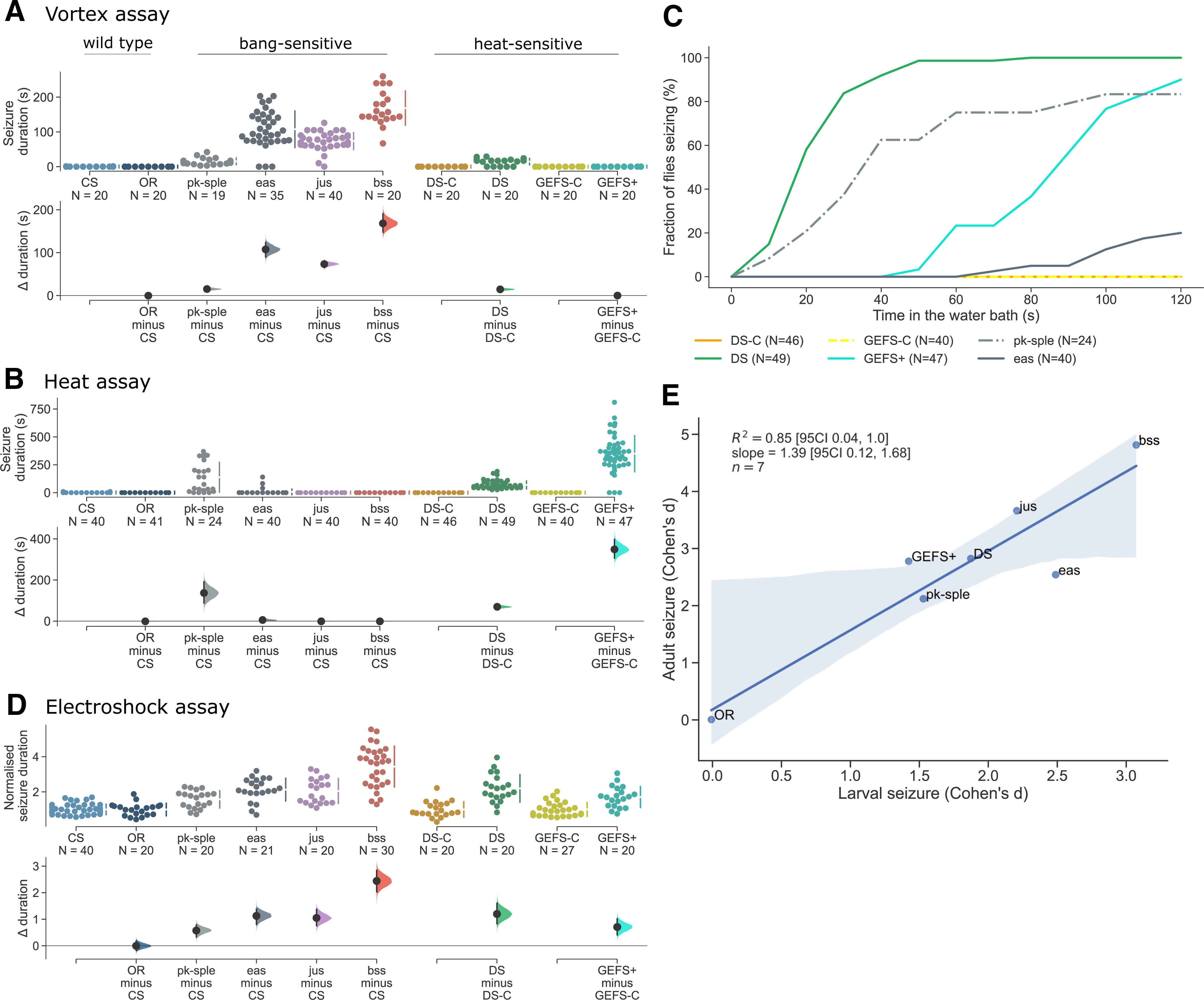
Comparison of seizure induction methods in *Drosophila* adults and larvae. We tested BS *pk-sple*, *eas*, *jus*, and *bss* lines and heat-sensitive DS and GEFS+ lines. ***A***, Vortex-induced seizures in *pk-sple* 15.22 s [95% CI: 11.37, 20.69], *eas* 107.54 s [95% CI: 88.71, 123.84], *jus* 73.35 s [95% CI: 64.60, 79.88], and *bss* 168.35 s [95CI: 157.31, 179.39]. No SLA was observed in CS, DS-C, DS, GEFS-C, and GEFS+ flies. ***B***, Seizure duration induced by the heat assay. DS showed seizures of average 71.82 s and GEFS+ of 431.03 s, with no seizures recorded for the respective matched controls (DS-C and GEFS-C). *pk-sple* showed seizures of 136.67 s [95% CI: 87.42, 191.84]. By comparison, CS, OR, *jus*, *bss*, or *eas* did not show heat-induced seizures. ***C***, Cumulative fraction of flies seizing throughout the 120 s of heat assay. Only 100% of DS flies seized during the 2-min period. Other genotypes reached a maximum of: 90% for GEFS+, 83% for *pk-sple*, and 20% for *eas*. ***D***, Electroshock induced seizures in all genotypes tested. Results of OR, *jus*, *eas*, *bss*, and *pk-sple* are reported as a ratio to CS seizure duration which was measured at 97.0 s [95% CI: 95.4, 101.6]. There were no differences between OR and CS. *Pk-sple* exhibited the weakest phenotype with seizure duration increased by 57.47%, whereas *bss* showed an increase of 244.11%. *eas* and *jus* had 112.62% and 104.95% longer seizures. DS and GEFS+ seizure durations are reported as a ratio to their respective controls (DS-C and GEFS-C). DS showed 119.78% and GEFS+ 70.67% increase in seizures as compared with controls. ***E***, Seizure induction in larva and adult flies have similar effectiveness (*R^2^* = 0.85 [95% CI: 0.03, 1.0], shaded area indicates 95% CI for the linear regression model fit). Effect sizes for adults were derived from vortex assay for BS and heat assay for temperature-sensitive lines.

*Drosophila* mutants with increased susceptibility to mechanical shock are known as BS (Parker et al., 2011a). There are more than a dozen BS single gene mutants (Baines et al., 2017), including para*^bangsenseless^* (*bss*) a hyperactive Na_v_ channel (Parker et al., 2011b), *easily-shocked* (*eas*) an ethanolamine kinase (Pavlidis et al., 1994), *julius seizure* (*jus*; also known as *slamdance*) with unknown protein function (Horne et al., 2017; Zhang et al., 2002), and *prickle^spiny legs^*(*pk-sple*), a protein involved in microtubule polarity (Tao et al., 2011; Ehaideb et al., 2014). Of these mutants, *bss* has the most severe seizure phenotype (Parker et al., 2011b). In our experiments with vortex induction (Fig. 1*A*), *bss* similarly exhibited the longest-duration seizures (168.4 ± 11.0 s; Fig. 2*A*). By comparison, *eas* exhibited an average seizure duration of 107.7 ± 8.9 s and *jus* of 73.6 ± 3.9 s. The majority of *pk-sple* did not exhibit severe seizures.

Identical stimulation of wild-type lines (CS, OR) resulted in only brief disruption to posture, with a typical recovery of 0.2 ± 0.1 s (Fig. 2*A*). Both heat-sensitive mutants, GEFS+ or DS, did not exhibit any seizure-like behavior following mechanical stimulation (Fig. 2*A*).

### Temperature seizure induction

Several seizure mutants are inducible by elevated temperature (Salkoff and Kelly, 1978; Kasbekar et al., 1987; Burg and Wu, 2012; Hill et al., 2019). Significantly, there are two lines which model-specific human epilepsy mutations: GEFS+ (Sun et al., 2012) and DS (Schutte et al., 2014). These “humanized knock-in” mutants exhibit seizures after exposure to elevated temperatures of 38°C and higher (Schutte et al., 2014). We tested the same range of mutants in heat-shock (Fig. 2*B*,*C*) as described for mechanical-shock induction, finding that 90% of GEFS+ flies exhibit seizure after 120 s, while 100% of DS flies exhibit seizure after 50 s at 41°C (Fig. 2*C*). Seizure duration, measured after the heat application was terminated, revealed seizures of average 349 s for GEFS+ and 72s for DS (Fig. 2*B*). This shows that although DS has a lower threshold for seizure induction, the seizure event is less severe compared with GEFS+.

Some BS lines have been shown to have seizures on exposure to cold temperatures including *bss* and *eas* at 8°C, but not at 39°C (Burg and Wu, 2012). We find that *eas*, *jus*, and *bss* do not exhibit seizures in the heat assay (at 41°C; Fig. 2*B*). By comparison, 83% of pk-sple tested showed seizure-like behavior with an average time of recovery around 137 s (Fig. 2*B*,*C*).

### Larval electroshock seizure induction

Larvae of *Drosophila* have a peristaltic motor action that has been exploited to study seizure. Application of a brief electric shock to the dorsal cuticle induces SLA (Marley and Baines, 2011; Fig. 1*C*). We tested whether this seizure-induction method could be used on both the mechanical-induced and heat-induced seizure mutants tested above. We found that, in response to electroshock, all the mutants exhibited long-lasting seizures (Fig. 2*D*). Of these, *bss* mutants exhibited the strongest phenotype having seizures 344% longer in duration than wild type (CS). Additional mutants, *jus* and *eas*, showed similar duration seizures at 105% and 113% longer than CS, whereas pk-sple seizures were 58% longer. Relative to their own genetic controls, GEFS+ and DS had seizure durations extended by 71% and 120%, respectively (Fig. 2*D*).

### Seizure induction method comparison

Since electroshock is applied at a different stage of development (i.e., larval) as compared with mechanical and heat-shock (i.e., adult), we compared efficacy of seizure induction methods across the different stages. Since larval and adult seizure duration metrics with different scales were being compared, rather than raw differences, we used Cohen’s *d*, a standardized effect size (Cumming and Calin-Jageman, 2016). Cohen’s *d* for adults was derived from the vortex assay on BS mutants and heat assay on HS mutants. They were compared with the efficacy of e-shock in larvae (Fig. 2*E*). The higher the value of effect size the stronger seizure phenotype was observed in the mutant as compared with control. Although the seizure assay at the adult stage had slightly bigger effect size than at the larval stage, there is a good correlation between the efficacies of seizure induction across the two different stages. This means that seizure severity across the two stages, larvae and adult, is comparable. Thus, electroshock seems to be an efficient and easily applicable method to induce reliable seizures in both mechanical-induced and heat-induced mutants, making it a favorable method to compare seizure severity across many, if not all, seizure mutations.

### Changes in adult locomotor activity

**Figure 3.**
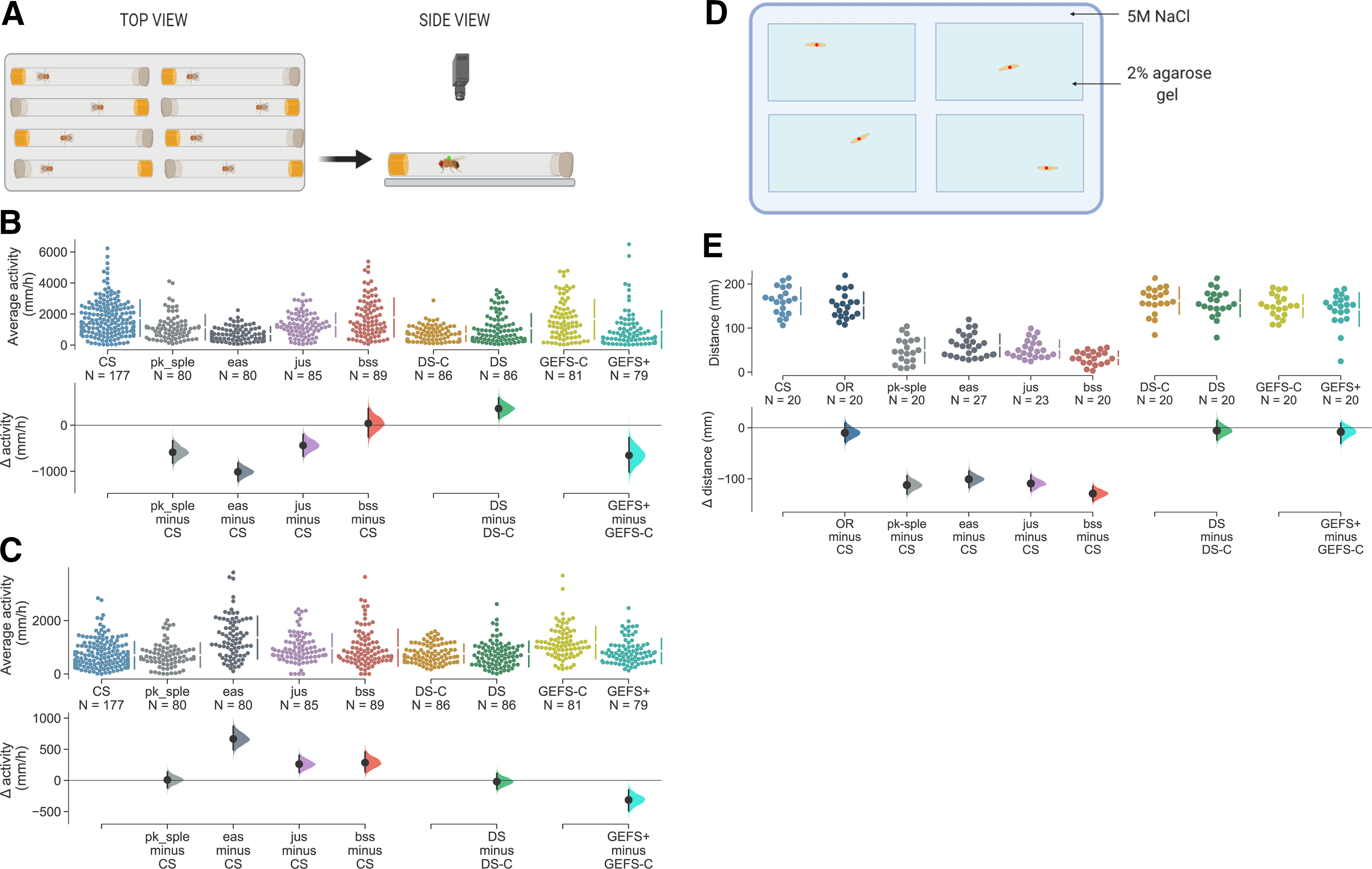
Adult seizure mutants do not exhibit obvious changes in locomotor activity. ***A***, Schematic showing the locomotor assay. ***B***, During the 12-h period of lights on, *jus* (1298.14 mm/h), *eas* (724.40 mm/h), *pk-sple* (1148.98 mm/h), and GEFS+ (1007.61 mm/h) show reduced locomotor activity (compared with appropriate controls), with *eas* and GEFS+ having the most prominent phenotypes; slower by 1012.09 mm/h [95% CI: −1211.17, −818.82] and 654.49 mm/h [95% CI: −1017.36, −267.84], respectively. DS showed increased activity by 361 mm/h [95% CI: 154.58, 592.13] over its control (DS-C) with an average speed of 727.60 mm/h. ***C***, During lights off, several mutants showed a small increase in activity levels. Of these, *eas* had the most prominent phenotype at 667.02 mm/h [95% CI: 493.03, 870.27]. *jus* and *bss* showed similar activity levels with an increase of 261.51 mm/h [95% CI: 128.67, 404.60] and 285.95 mm/h [95% CI: 138.50, 458.49], respectively. GEFS+ is the only genotype remaining slower than its respective control (GEFS-C) by 313.14 mm/h [95% CI: −489.98, −154.34]. Other genotypes showed no meaningful differences. Fly activity was tracked for 3 d at 25°C at 12/12 h light/dark cycle. All genotypes were recorded at least twice. ***D***, Schematic showing the set up for larval tracking. ***E***, Total distances crawled by BS mutants *pk-sple* (49.27 mm), *eas* (60.51 mm), *jus* (52.29 mm), and *bss* (32.43 mm) were greatly lower in comparison to CS (161.47 mm). Heat-sensitive mutants did not show any meaningful reduction in the total distance crawled from their matched controls.

Alterations in activity patterns and/or sleep duration have been reported for *Drosophila* seizure mutants (Petruccelli et al., 2015). Some epilepsy patients, including DS sufferers present with associated ataxias (Oakley et al., 2011; Marcián et al., 2016). We hypothesized that fly seizure mutants could have similar secondary phenotypes in addition to seizure, and that these may provide “simpler” predictors of seizure susceptibility. To address this, we measured adult locomotor activity over a 3-d period (12/12 h cycle) using a custom video-tracking system (Fig. 3*A*). During the light phase (subjective day) BS mutants showed slightly lower activity (Fig. 3*B*). During darkness (i.e., subjective night) the same seizure-mutant flies tended to have an increased activity compared with wild-type controls (Fig. 3*C*). The largest difference from CS wild type during subjective day was exhibited by *eas* at 1012 mm/h reduction, the largest difference during subjective night was also exhibited by *eas,* where it was more active by 667 mm/h.

Of the heat-inducible lines, GEFS+ was slower by 654 mm/h during the day and by 313 mm/h during night relative to its own matched genetic control. However, DS was 361 mm/h more active in light but showed no difference to the controls in darkness. Out of the lines investigated in this study, DS is the only genotype to have increased activity during the day. GEFS+ was the only genotype to be consistently slower than control.

The stark differences between day-phase and night-phase activity changes for the differing mutants tested, and the absence of a consistent pattern between activity changes and seizure-induction type or severity, indicate the hypothesis is false: adult locomotor activity is not a reliable predictor of seizure severity.

### BS mutants show reduced larval locomotion

There are previous reports showing alterations in either larval locomotion or peristaltic wave frequency in seizure susceptible *Drosophila* mutants (Stilwell et al., 2006; Graham et al., 2016; Streit et al., 2016). We decided to test for such in the range of mutants used for this study and compare that to the results of adult activity. We performed larval tracking and reported a total distance crawled by each genotype in a 3-min period (Fig. 3*D*,*E*). CS wild type had the most activity with a total distance traveled of 161.47 mm. The range of BS mutants showed a consistently lower locomotion level as compared with CS: *pk-sple* slower by 112.20 mm [95% CI: −129.94, −94.07], *eas* by 100 mm [95% CI: −117.24, −84.80], *jus* by 109.18 mm [95% CI: −124.02, −92.49], and *bss* being the slowest with a reduction of 129.05 mm [95% CI: −143.67, −114.46]. Unlike BS mutants, the range of HS flies did not show any meaningful differences in their locomotor activity among themselves, and in comparison to CS (Fig. 3*E*). These differences in phenotype reinforce the observation from adult fly activity levels, levels of larval locomotor activity are also not predictive of seizure severity.

### Larval motor neuron activity patterns are not predictive of seizures

**Figure 4.**
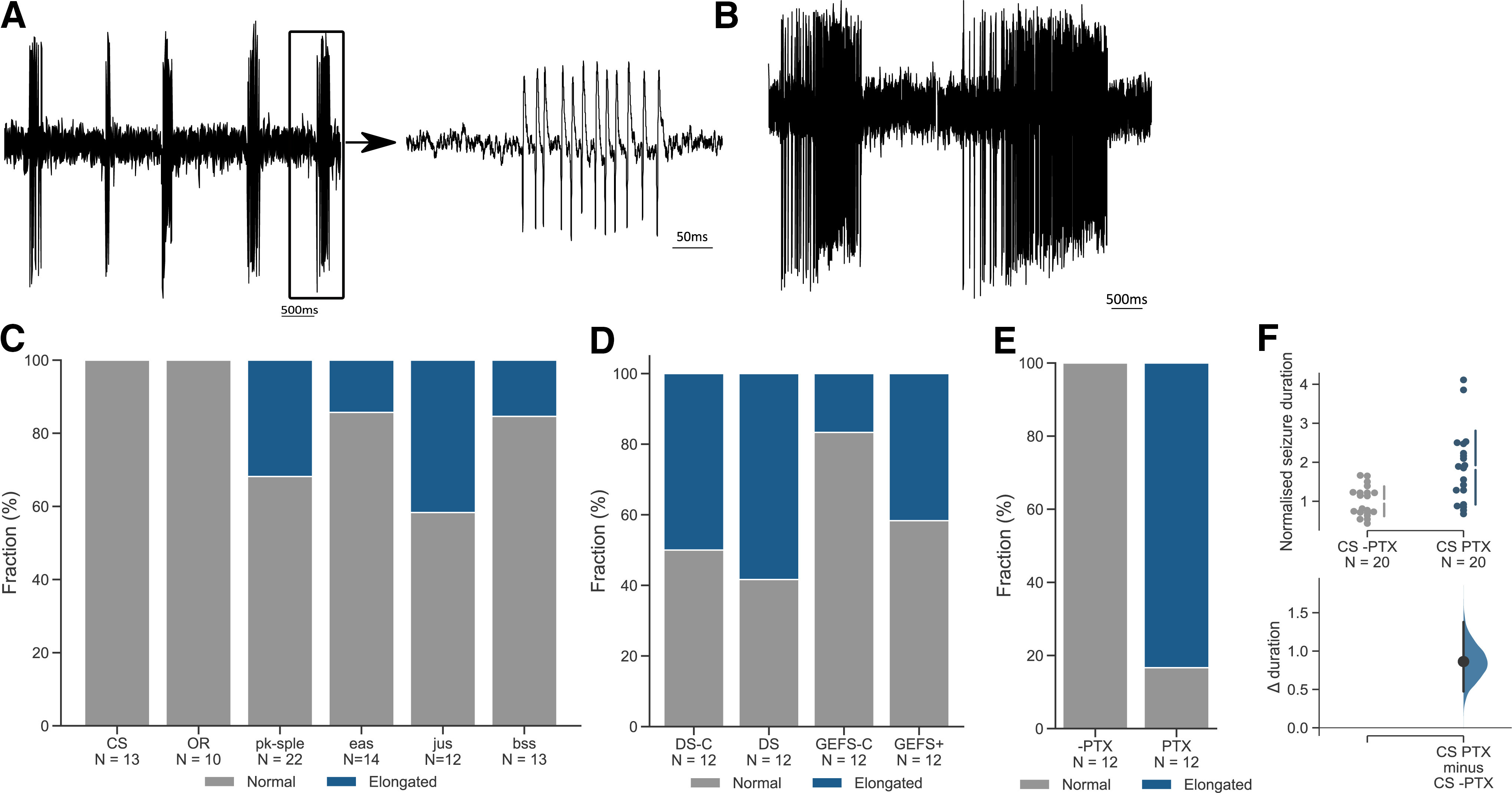
Seizure mutants show elongated motor neuron bursting. ***A***, ***B***, Examples of cell-attached recordings from larval RP2 and aCC motor neuron bursting patterns in CS wild-type (***A***) and the *bss* seizure mutant (***B***). ***C***, The fraction of elongated bursts (i.e., activity shown in ***B***) observed in wild-type and the BS genotypes: CS, OR (0%), *pk-sple* (31.8%), *eas* (14.29%), *jus* (41.67%), and *bss* (15.38%). ***D***, Fraction of elongated bursts recorded in heat-sensitive mutants and their respective controls: GEFS-C (16.67%), GEFS+ (41.67%), DS-C (50.00%), and DS (58.33%). ***E***, Fraction of elongated bursts detected in CS larvae fed with either vehicle (–PTX) or PTX. Vehicle showed only normal bursting, but recordings from PTX-fed larvae showed 83.33% elongated bursts. ***F***, PTX induces larval seizures with recovery time 86% longer in the experimental group than control.

Observing that larval electroshock applies to the full panel of seizure mutants we have tested, we decided to investigate electrophysiological properties that may be associated with seizure susceptibility. Because the behavior we observed is because of altered muscular activity, we focused attention to motoneurons. We performed loose patch recordings from the aCC or RP2 motoneurons in third instar larvae. These motoneurons were selected because of their accessibility and prior extensive characterization (Baines and Bate, 1998; Choi et al., 2004). Recordings from aCC/RP2, in wild-type larvae, show a spontaneous, identical and robust bursting pattern of action potentials (APs) which represents the output of the locomotor central pattern generator (Baines et al., 1999; Fig. 4*A*). However, alterations in burst recordings were noticed in BS mutants (Fig. 4*B*). To characterize bursting, we used spike times from recordings of 180 s. We defined a burst as an event with a minimum of three spikes occurring within 100 ms from one another which ends when no spike is detected within the following 100 ms. Based on this classification, recordings from aCC/RP2 from wild-type CS or OR showed one type of bursting pattern (termed “normal”) where bursts have an average of 14 or 16 APs, respectively (Table 1). Using the same data, we also gathered average burst duration SD, burst frequency, AP count per burst and frequency per burst (Table 1). The data revealed that although there is some variation in all of these parameters, burst duration is consistent with relatively small variation in wild-type but larger variation (in terms of observed SD) in seizure mutants (Table 1). Based on this insight, we classified bursting traces as normal if the average burst duration was the mean of CS burst ± 1.5 SD. Using this classification, we found all mutant lines tested had some normal bursting in addition to some elongated bursting traces (Fig. 4*C–E*). The fraction of elongated bursting was variable across genotypes, but consistently observed in both BS and HS seizure mutants. Of the mutants tested, *jus* had the highest fraction of elongated bursts among BS (42%; Fig. 4*C*) and DS among the HS mutants (58%; Fig. 4*D*). However, unlike the two wild types, DS and GEFS+ matched genetic controls also showed appreciable elongated burst firing. Thus, compared with their respective controls (and not true wild types), DS and GEFS+ showed increased elongated bursting of 17% and 150%, respectively. This, however, suggests that elongated bursting is not exclusive to seizure mutants.

**Table 1:** Analysis of activity recordings from larval motor neurons

Genotype	*N*	Burst duration (ms)	Burst duration SD (ms)	Burst frequency (Hz)	Firing rate (AP/s)	AP count per burst (AP/burst)	AP frequency per burst (Hz)
CS	13	174.57	91.54	0.47	6.68	14.25	87.53
OR	10	155.40	84.80	0.40	6.38	16.24	104.30
*pk^sple^*	*22*	394.01	348.54	0.46	6.92	16.81	51.48
*jus*	*12*	703.94	1211.46	0.31	8.23	23.26	58.88
*eas*	*14*	210.22	165.49	0.49	6.73	12.78	69.78
*bss*	*13*	195.42	215.28	0.43	4.97	12.72	77.89
*GEFS-C*	*12*	208.94	183.28	0.37	4.32	13.67	76.16
*GEFS+*	*12*	1160.34	1827.30	0.46	9.48	49.54	362.66
*DS-C*	*12*	11,859.25	11,869.45	0.39	6.21	166.21	322.43
*DS*	*12*	3139.83	4338.58	0.23	4.39	42.26	34.13
*CS* –*PTX*	*12*	170.16	89.59	0.30	4.07	12.59	90.34
*CS +PTX*	*12*	1964.82	5369.33	0.37	13.99	65.30	40.28

Three-minute loose-patch recordings of spiking activity were analyzed using a custom-built MATLAB script. Bursts were defined as three or more spikes occurring within 100 ms from each other. The numbers are averages for all traces per genotype.

PTX is known to induce SLA in wild-type flies. The advantage of this approach is that the genetic background is identical between control and PTX-fed animals. We tested for elongated bursting in a PTX-induced seizure model (CS fed PTX) and found that the fraction of elongated bursting was increased to 83%. By contrast unfed control animals showed no elongated bursts (Fig. 4*E*). PTX-induced seizures in larvae were verified using the e-shock assay, results of which show an increase in recovery time by 86% (Fig. 4*F*).

**Figure 5.**
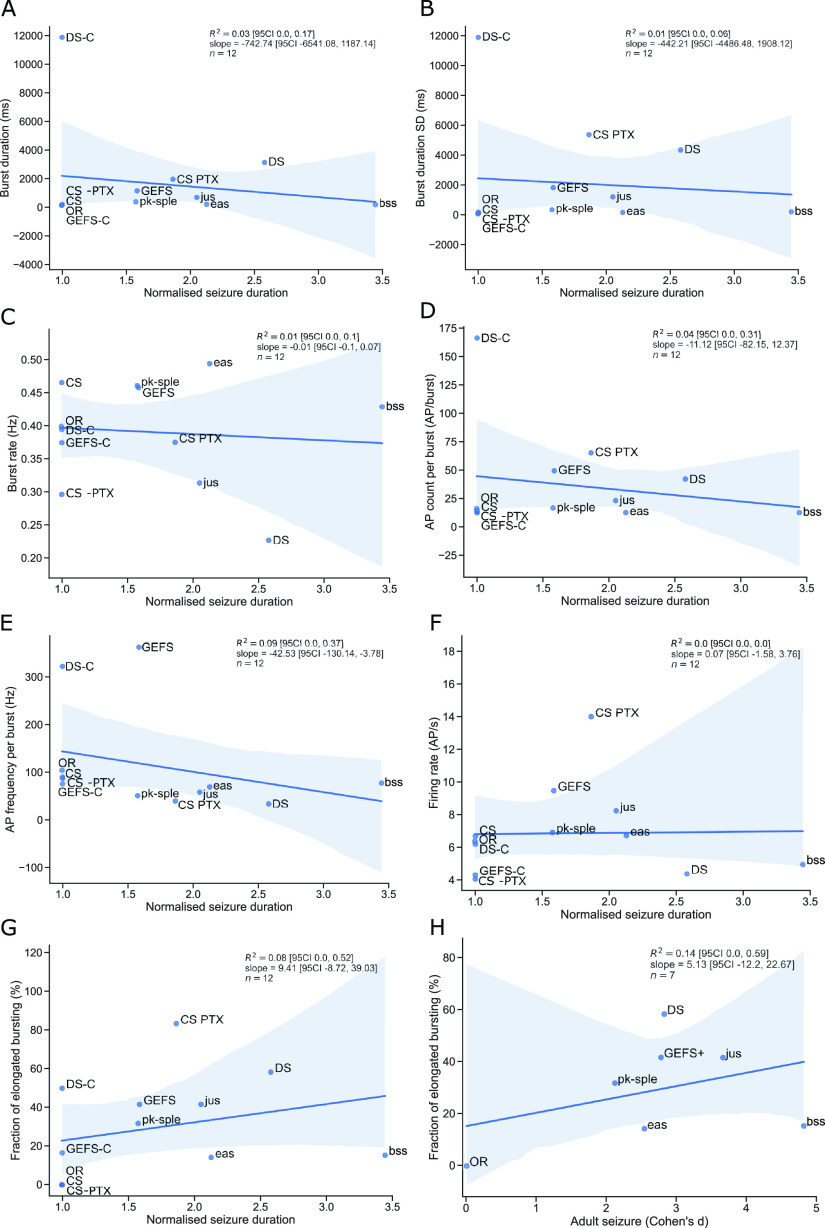
Larval motor neuron firing does not predict seizure susceptibility. Analysis of 3-min loose-patch recordings from larval RP2/aCC motor neurons revealed that there is no correlation between mean burst duration (***A***), SD of burst duration (***B***), burst frequency (***C***), AP count per burst (***D***), AP frequency per burst (***E***), AP firing rate (***F***), and fraction of elongated bursting (***G***) and larval seizure. (***H***) Fraction of elongated bursting from larval motor neurons showed no correlation with the effect size of adult seizure. Shaded areas indicate 95% CI for the linear regression model fit.

We further investigated how aberrations in bursting recordings might correlate with larval seizure (Fig. 5). We used mean burst duration, burst duration standard deviation, burst frequency, AP firing rate, AP per burst count and frequency. None of these parameters revealed any correlation between them and larval seizure severity (Fig. 5*A–F*). However, frequency with which abnormalities occur could have a more significant impact on seizure susceptibility than the absolute values of deviations in burst duration. We investigated whether the fraction of elongated bursting could be predictive of seizure duration. We found that there is no strong correlation (*R*^2^ = 0.08) between the two measures (Fig. 5*G*). The alterations in larvae development could potentially be predictors of adult phenotypes, however, when we compared adult seizure to bursting patterns, the measures were also not well-correlated (*R*^2^ = 0.14; Fig. 5*H*). Thus, we conclude that while altered motor neuron activity is typical for seizure mutants, it is not diagnostic for seizure severity.

## Discussion

Epilepsy is characterized by recurrent seizures, which can manifest as absence seizures, underlying periods of inattention, through to full generalized myoclonic seizures, resulting in muscle jerks to full collapse and unconsciousness. While *Drosophila* seizure mutants lack these full range of behaviors, seizures in both humans and flies nevertheless exhibit sufficient parallels to implicate that the underlying neuronal abnormalities are highly similar. Previous investigations have shown in *Drosophila* that seizures include (1) a defined seizure threshold, (2) genetic mutations that modify seizure-susceptibility, (3) electroshock therapy raises the threshold for subsequent seizures, (4) seizures spread throughout the CNS along defined neuronal tracts, (5) seizures can be localized to distinct regions of the CNS, and (6) seizures can be ameliorated by AEDs used to treat human epilepsy (Kuebler and Tanouye, 2000; Kuebler et al., 2001; Reynolds et al., 2004; Tan et al., 2004; Marley and Baines, 2011; Lin et al., 2017). Unlike human epilepsy, however, fly seizure mutants do not routinely exhibit spontaneous seizures which may reflect fewer numbers of neurons within the latter. However, there are a couple of notable exceptions: pk-sple (Tao et al., 2011; Ehaideb et al., 2016) and DS (Schutte et al., 2014). Nonetheless, seizures in flies can be successfully induced using either mechanical, temperature or electroshock induction in a variety of single gene mutant backgrounds, much as seizures can be induced in mammals by extreme external stimuli (light or sound), fever, and/or electroshock.

Our study reproduced earlier results validating that all of the lines tested exhibit induced-seizure behavior by a variety of methods (Pavlidis et al., 1994; Zhang et al., 2002; Parker et al., 2011a; Tao et al., 2011; Sun et al., 2012; Schutte et al., 2014; Horne et al., 2017). The length of seizure varies by mutation, with *bss* being the strongest BS phenotype in our experiments and, indeed, the strongest reported so far (Parker et al., 2011b). GEFS+ exhibited longer duration seizures than DS following heat-shock, although DS had a lower seizure induction threshold. This is similar to humans, where DS is normally a more severe syndrome than GEFS+ (Brunklaus and Zuberi, 2014).

As in adults, seizures can be induced in *Drosophila* larvae. Electroshock induction has been successfully applied before (Marley and Baines, 2011). These authors reported the *sda* mutant larva (renamed to *jus*; Horne et al., 2017) exhibit seizure durations up to 6.6 times longer than CS wild-type controls. Likewise, our electroshock induction was effective in inducing seizures in all mutants studied. Seizure duration was variable by genotype, with the longest duration seizures seen in *bss*. Comparing the severity of seizures induced in either adults or larvae suggests that, although adults tend to have longer and more complex seizures, the chosen method for induction is critical.

Larval electroshock is comparable to adult electroshock. Adult electroshock successfully induces seizures in a range of BS mutants including *eas*, *bss*, and *jus* tested in our study (Pavlidis et al., 1994; Pavlidis and Tanouye, 1995). There is a stereotypical sequence of seizure in adults including two distinct phases of dorsal longitudinal muscle failure defined as a silent period lasting 35–40 s and abnormal responses during recovery which varied by genotype. However, adult electroshock does not always evoke seizures in temperature-sensitive mutants, e.g., *seizure*. By contrast, our results indicate that electroshock of larvae successfully induced seizures in all mutants and, moreover, generated a comparable hierarchy of seizure severity to adults. Similar to adult electroshock, the response is quite stereotyped in stages. It starts with larval paralysis of varied duration depending on genotype. This is followed by both, or either, partial peristaltic waves and mouth hook contractions of high frequency. Recovery is determined once several full peristaltic waves occur in the animal (Marley and Baines, 2011).

There are other methods available to reliably induce seizures in many mutants such as the proconvulsant PTX, which works in both larvae and adults (Stilwell et al., 2006; Lee et al., 2019). However, in this study we show that electroshock is a simple and reliable method, applicable to many seizure genotypes. We would suggest its use for studies of novel gene mutations considered to be seizure related.

We also explored whether adult seizure mutants exhibit pronounced alterations in locomotion, without seizure induction, as has been reported in some human epilepsies (particularly those associated with ataxias). Tracking adult fly activity over several days revealed that there are changes in daytime activity, although these were not consistent across the genotypes studied. During the night, activity was increased; this partly mimics human disorders where epilepsy impairs sleep (Jain and Kothare, 2015; Wang et al., 2018). However, there was again no obvious uniform trend. As such, our results only partially replicated previously observed changes in activity (Petruccelli et al., 2015), whereas the previous study shows an increase in activity during the night, we observed an overall decrease in GEFS+ activity in both day and night. This might be explained by accumulation of genetic modifiers over time. Alterations in food composition are also known to affect seizure phenotype in flies and potential differences in fly food could also impact secondary phenotypes observed (Stone et al., 2013; Fogle et al., 2019; Kasuya et al., 2019). On the other hand, the overall trend we observed matches previously reported data where seizure mutants are, overall, less active than controls (Radlicz et al., 2019).

Earlier research suggests that the reduced levels of sleep in flies could lead to increased seizure susceptibility (Lucey et al., 2015). The study showed that, in seizure prone BS flies *ses^B^* and heat-sensitive *sei^TS^*, there are no alterations to sleep pattern, but sleep deprivation leads to increased seizure severity. However, another study by Petruccelli et al. (2015) reported reduced seizure likelihood and increased seizure threshold after sleep deprivation in GEFS+ flies. Clearly, fly models of epilepsy (like in human patients) are heterogenous and each mutation potentially has a distinct relationship to sleep and its effect on susceptibility to seizure. Both prior research and the present study suggest that sleep–seizure relationships could be investigated further for each genotype individually to better understand the underlying mechanisms.

Changes in larval locomotion exhibited a clear difference between mechano-sensitive and heat-shock-sensitive lines not evident in adult behavior. BS larvae showed a consistently lowered activity whereas HS larvae did not show any movement phenotype. A previous report shows that both GEFS+ and *bss* exhibit an increase in synchronicity in larval peristaltic waves (Streit et al., 2016); however, in our study, it did not translate to the same extent of alterations to locomotion. Moreover, there does not seem to be a clear relationship between locomotion and recovery time from electroshock in larvae. BS lines like *pk^sple^*, *jus*, and *eas* have reduced locomotion but their recovery time is comparable to DS and GEFS+ which display higher levels of locomotive activity.

Susceptibility to seizures may arise from mutations in a diverse range of genes including those that encode ion channels and/or microtubule polarity-associated genes. However, given a similar behavioral phenotype (i.e., seizure), we explored the possibility of a common underlying change in cellular activity. To investigate this idea, we performed loose-patch recordings from larval motor neurons aCC or RP2 (note, these two neurons are highly similar in their biophysical properties; Baines et al., 1999). In wild-type larvae (CS and OR), these neurons exhibit a robust and consistent bursting pattern. The same activity patterns were also present in the seizure mutants and their matched controls. However, in addition, seizure mutants also exhibited altered activity patterns that were not observed in the wild types. This altered activity showed increased burst duration accompanied by modest increases in AP firing frequency. Increased AP firing is a hallmark of epileptic seizures (Marley and Baines, 2011; Parker et al., 2011b; Ehaideb et al., 2016). However, our data clearly shows that the presence of elongated burst firing is not well-correlated to induced seizure duration. Thus, at present it is difficult to understand the causes of altered firing activity. The fact that such activity was also observed in both GEFS+ and DS control lines are suggestive that it is not exclusive to seizure mutants. To negate differences in genetic background, that may complicate our results, we fed PTX to CS and observed a greatly increased fraction of elongated bursting. Thus, while burst elongation might not be a sufficient explanation for seizure severity, these prolonged activity bursts are clearly associated with seizure per se. It is possible that the poor correlation with seizure severity could be because of differing efficiencies of neuronal homeostatic compensation present in the differing seizure mutations we investigated.

In conclusion, *Drosophila* seizure mutants form a diverse group of models of epilepsy syndromes. Mutants can be separated by seizure induction modes; of the methods tested here, larval electroshock is the induction method most broadly applicable. The more general adoption of larval electroshock could help reduce variability between reported studies that use differing methods of seizure induction. Thus, we recommend this method as a benchmark technique for testing known and novel *Drosophila* seizure mutations.

## Synthesis

Reviewing Editor: Deanna Smith, University of South Carolina Decisions are customarily a result of the Reviewing Editor and the peer reviewers coming together and discussing their recommendations until a consensus is reached. When revisions are invited, a fact-based synthesis statement explaining their decision and outlining what is needed to prepare a revision will be listed below. The following reviewer(s) agreed to reveal their identity: Atulya Iyengar, David Deitcher.

Both reviewers find value in the work, indicating that conceptually, cross-analysis of drosophila epilepsy mutants is an important reference for the field that will accelerate progress by showing the utility of larval electroshock as a reliable method for assaying seizure mutants. While the reviewers do not propose new experiments, they had some concern about the reported high-temperature sensitivity of bss1 and would like to see the experiment repeated. There are other concerns about precise writing, appropriate referencing, and statistical methods. I have included the specific comments from each reviewer.

Reviewer 1:

The authors of this manuscript are interested studying/contrasting the seizure susceptibility across large collection of “fly seizure genotypes” using a standardized set of behavioral and electrophysiological methods. Epilepsy research in Drosophila utilizes a wide array of behavioral protocols. Such a standardized analysis of epileptic phenotypes across Drosophila mutants would provide an important reference for the field. Although the goal of the manuscript is quite admirable, the results and interpretation come up short, and may be easily improved for clearer impact.

Introduction & General Points:

1) The manuscript suffers from imprecise language throughout the manuscript. An initial example can be found in the title: “Characterization of induction methods for Drosophila seizure mutations”. It is not clear what the authors are studying, methods to induce seizure mutations (i.e. gene editing)? Or new methods to induce seizures? The authors need to carefully review the manuscript to make sure they say what they mean to say.

2) The authors do not consider/discuss the large literature surrounding adult electroshock experiments. This quite surprising, because the technique is very well established and effective in inducing seizures in nearly all of the mutants examined in this manuscript (and many other not described here, e.g. BK slowpoke mutants, Ghezzi et al., 2010 PNAS).

3) Throughout the manuscript make claims like “There has not yet been a systematic analysis of the wide range of behavioral and physiological phenotypes observed across major fly seizure genotypes” (Abstract). This statement is not appropriate. Many reviews have been written comparing seizure phenotypes across mutants (e.g. Cunliffe et al., 2015; Parker et al. 2011 review). Additionally, Keubler, 2000, Keubler 2001, Lee & Wu 2006 systematically address how different excitability mutants modify electroconvulsive seizure repertoire (including many mutants used in this study). Although the authors cite Keubler et al., 2001, it is somewhat surprising that they do not recognize that Keubler et al., performed a systematic analysis of seizure threshold across a wide array of mutants.

4) The authors citations need to be done appropriately. Wherever possible, the authors should cite the original literature. Examples: Intro 3rd PP Parker et al., did not develop the electroconvulsive seizure protocol in flies. (Pavlidis et al., 1994; or Pavlidis & Tanouye 1995 is more appropriate). If the authors are referring to behavior seizure-like activity, or a comparison of behavior and electrophysiological repertoires it is more appropriate to cite Ganetzky & Wu 1982, and Lee & Wu, 2002)

5) “Reliable method to induce seizures” is somewhat misleading. Adult electroconvulsive (high-frequency) stimulation induces seizures in all kinds of mutants. Additionally, there are many proconvulsants (e.g. PTX, see Stillwell et al., 2006 for larvae, Iyengar & Wu, 2019 for adults) which will reliably induce seizures in most mutants. In vertebrates, PTZ, PTX, 4AP, Penicillin and high K can reliably induce epileptiform behavior. There is no reason to think either of these methods would be less reliable in inducing seizures.

6) “Seizure severity” (P4. Last sentence) How is this operationally defined? This needs to be clearly spelled out.

Results:

7) Figure 2: The authors should clearly mark which lines have been previously reported to be bang-sensitive, and which ones are not reported to be bang sensitive.

8) Figure 2: Several groups have previously reported bss is not high temperature sensitive (e.g. Burg & Wu, 2012). It appears that the Figure 2B results match previous findings apart from a few outliers that were “seizing” for 20 min. Before making the claim of temperature sensitivity, the authors should eliminate the possibilities that these outliers reflect either dead flies, or flies undergoing seizure due to mechanical handling.

9) Figure 2A-D: How were the distributions for delta duration computed? They don’t seem to match either the seizure duration (above) or a normal distribution?

10) Cohen’s D: It would be helpful to the reader if the authors discuss why this method was chosen (as opposed to other methods to measure “effect size”), and how it was parameter was computed (i.e. the formula). How would this parameter compare with the coefficient of variation (CV)?

11) Figure 2: In panel E, what is meant by adult seizure? Is this heat induced seizure or vortex-induced seizure?

12) Figure 2E: Why is it appropriate to perform a linear regression? What does that mean? How was the R2 value computed (each genotype is a point?)

13) Figure 3: The authors can be a bit more sophisticated in their thinking (“adult locomotor activity is not a reliable predictor of seizure severity”). Perhaps night activity by itself, or irregular activity patterns lead to susceptibility to seizures? There are several clinical disorders that affect circadian/sleep patterns and predispose patients to night-time seizures.

14) Figure 3: The finding of a consistent larval locomotion defect phenotype in bang-sensitive mutants is an important one. Does this affect recovery from larval electroshock?

15) Figure 5: The authors don’t completely discuss their data set. What does CS EtOH mean?

Discussion

16) One of the general conclusions of the paper is that larval electroshock is a “broadly applicable [seizure] induction method” and is a recommended assay for hyperexcitable mutants. How does the approach compare to electroconvulsive seizure methods applied in adults? In adults the timing of distinct phases of the electroconvulsive seizure discharge seizure sequence is quite stereotypic (e.g. the delayed seizure discharge onset is +/- 3 s following a ∼20 s paralysis phase).

Reviewer 2:

The manuscript nicely compares different seizure mutants with a large variety of assays to determine which assays reflect the underlying severity of the mutant. I believe that this careful work will accelerate the progress in the field by showing the utility of larval electroshock as a reliable method for assaying seizure mutants. Other work has looked at some of these methods but no paper has used so many methods and applied it to so many of these mutants.

The writing could be more precise and more credit should be given to earlier studies on characterizing different seizure-prone mutants. The authors should also address why they observe temperature sensitivity in the bss line while others have not seen this phenotype.

## Author Response

Response to Reviewers

Dear Editor

We are submitting our revision of 'Characterization of induction methods for Drosophila seizure mutations (original title) #eN-NWR-0079-21'. We thank the two reviewers for their helpful comments and describe how we have revised the manuscript as follows:

Reviewer 1

1. The manuscript suffers from imprecise language throughout the manuscript. An initial example can be found in the title: “Characterization of induction methods for Drosophila seizure mutations”. It is not clear what the authors are studying, methods to induce seizure mutations (i.e. gene editing)? Or new methods to induce seizures? The authors need to carefully review the manuscript to make sure they say what they mean to say.

We have changed the title to 'Characterisation of seizure induction methods for Drosophila'

2. The authors do not consider/discuss the large literature surrounding adult electroshock experiments. This is quite surprising, because the technique is very well established and effective in inducing seizures in nearly all of the mutants examined in this manuscript (and many others not described here, e.g. BK slowpoke mutants, Ghezzi et al., 2010 PNAS).

We have included a paragraph in the introduction to address this comment:

"There is a third, and more involved seizure induction method in adult flies, known as high-frequency stimulation of the giant fibre (GF) pathway (Pavlidis and Tanouye 1995). This method induces seizures in all bang-sensitive genotypes. Whilst this assay allows investigation of synaptic transmission during seizures, stimulation of the GF pathway requires a more complex set up than either the vortex or temperature-shock assays and is not suitable for medium- or high-throughput screening.”

3. Throughout the manuscript make claims like “There has not yet been a systematic analysis of the wide range of behavioral and physiological phenotypes observed across major fly seizure genotypes” (Abstract). This statement is not appropriate. Many reviews have been written comparing seizure phenotypes across mutants (e.g. Cunliffe et al., 2015; Parker et al. 2011 review). Additionally, Keubler, 2000, Keubler 2001, Lee & Wu 2006 systematically address how different excitability mutants modify electroconvulsive seizure repertoire (including many mutants used in this study). Although the authors cite Keubler et al., 2001, it is somewhat surprising that they do not recognize that Keubler et al., performed a systematic analysis of seizure threshold across a wide array of mutants.

To address this comment, we have included the sentences below in the abstract and introduction:

"To better understand behavioural and physiological phenotypes across major fly seizure genotypes, we systematically measured seizure severity and secondary behavioral phenotypes at both the larval and adult stage.”

"Several reviews have previously characterised fly electrophysiological and behavioural responses to specific seizure assays (Kuebler and Tanouye 2000; Lee et al. 2019; Parker et al. 2011; Pavlidis and Tanouye 1995).”

4. The authors citations need to be done appropriately. Wherever possible, the authors should cite the original literature. Examples: Intro 3rd PP Parker et al., did not develop the electroconvulsive seizure protocol in flies. (Pavlidis et al., 1994; or Pavlidis & Tanouye 1995 is more appropriate). If the authors are referring to behavior seizure-like activity, or a comparison of behavior and electrophysiological repertoires it is more appropriate to cite Ganetzky & Wu 1982, and Lee & Wu, 2002)

We thank the Reviewer for the corrections, we have updated the references in the introduction to cite original studies.

5. "Reliable method to induce seizures” is somewhat misleading. Adult electroconvulsive (high-frequency) stimulation induces seizures in all kinds of mutants. Additionally, there are many proconvulsants (e.g. PTX, see Stillwell et al., 2006 for larvae, Iyengar & Wu, 2019 for adults) which will reliably induce seizures in most mutants. In vertebrates, PTZ, PTX, 4AP, Penicillin and high K can reliably induce epileptiform behavior. There is no reason to think either of these methods would be less reliable in inducing seizures.

We found electroshock to be a reliable method to induce seizures across a range of mutations that we have tested. This does not contradict the fact that other methods whether adult electroshock or chemicals are also reliable and applicable.

6. “Seizure severity” (P4. Last sentence) How is this operationally defined? This needs to be clearly spelled out.

To clarify this, we have edited the statement as below (page 4):

"However, the alteration was not exclusive to seizure mutants and did not have good predictive power for seizure severity measured as seizure duration.”

Results:

7. Figure 2: The authors should clearly mark which lines have been previously reported to be bang-sensitive, and which ones are not reported to be bang sensitive.

We thank the Reviewer for this suggestion and now clearly state this point in Figure 2 legend and have added new labels to Figure 2a.

8. Figure 2: Several groups have previously reported bss is not high temperature sensitive (e.g. Burg & Wu, 2012). It appears that the Figure 2B results match previous findings apart from a few outliers that were “seizing” for 20 min. Before making the claim of temperature sensitivity, the authors should eliminate the possibilities that these outliers reflect either dead flies, or flies undergoing seizure due to mechanical handling.

We have now repeated the heat assay twice with bss animals aged 5 days (which is more similar to the age of animals used for the other genotypes), the original data was from adults aged 10 days or more. We did not observe any seizures in these repeat experiments and data in Figure 2B has been updated accordingly. It is interesting that previously used older animals (>10 days old) could explain the difference, although at this time we have chosen not to further investigate this relationship. We thank the reviewer for the astute observation.

9. Figure 2A-D: How were the distributions for delta duration computed? They don’t seem to match either the seizure duration (above) or a normal distribution?

The distributions were calculated using the bootstrap method, as implemented in the DABEST package (Ho et al. 2019); this software is suggested by eNeuro for visualizing effect sizes (Bernard 2019). The distributions shown are of the 5000 mean differences calculated in the bootstrap resampling procedure. As predicted by the central limit theorem, the mean-difference distributions are more normal (though typically not perfectly normal) than either of their corresponding observed-value distributions.

The text below was included in Materials and Methods - Statistical analysis - section to jointly answer this and the following question:

"The effect size distributions shown are of the 5000 mean differences calculated in the bootstrap resampling procedure. To compare effect sizes across different assays a universal effect size measure Cohen’s d was used. Cohen’s d is an effect size where the difference has been standardized to the standard deviations of the two groups being compared.”

10. Cohen’s D: It would be helpful to the reader if the authors discuss why this method was chosen (as opposed to other methods to measure “effect size”), and how the parameter was computed (i.e. the formula). How would this parameter compare with the coefficient of variation (CV)?

Cohen’s d is an effect size where the difference has been standardized to the standard deviations of the two groups being compared. Since larval and adult seizure duration metrics with very different scales were being compared, rather than raw differences, we used a universal standardized metric for effect size comparisons. Though not essential, this does follow convention from meta-analysis, where diverse metrics are incorporated after transformation into d. Although the coefficient of variance is also a standardized metric, it is not an effect size.

11. Figure 2: In panel E, what is meant by adult seizure? Is this heat induced seizure or vortex-induced seizure?

The results section states that adult seizure effect size was measured using vortex assay for mechano- and heat-shock assay for heat-sensitive mutants. To make this more explicit in Figure 2, we introduce this explanation in Figure 2E caption:

"Effect sizes for adults were derived from vortex assay for bang-sensitive and heat assay for temperature-sensitive lines.”

12. Figure 2E: Why is it appropriate to perform a linear regression? What does that mean? How was the R2 value computed (each genotype is a point?)

We inferred a linear relationship based on the scatter plot produced and tested the fit of the linear model by calculating R2. The line was fitted and R2 was calculated using the linregress function in Python library “scipy", which finds the line with the least-squares regression. Effect sizes from each assay were converted to a universal effect size measure Cohen’s d and calculated for each genotype. Each genotype represents a point. The result suggests that there is a strong linear relationship between the seizure severity in larvae and adults, both of developmental stages show similar levels of susceptibility to seizure and its duration.

The below text has been included in the Materials and Methods section:

"Inferred linear relationships between various parameters were assessed using linear regression. The line was fitted and R2 was calculated using scipy library in Python using linregress function, which finds the line with the least-squares regression. Each point represents a genotype.”

13. Figure 3: The authors can be a bit more sophisticated in their thinking (“adult locomotor activity is not a reliable predictor of seizure severity”). Perhaps night activity by itself, or irregular activity patterns lead to susceptibility to seizures? There are several clinical disorders that affect circadian/sleep patterns and predispose patients to night-time seizures.

To address the comment we have included the paragraph below in the discussion:

"Earlier research suggests that the reduced levels of sleep in flies could lead to increased seizure susceptibility (Lucey et al. 2015). The study showed that, in seizure prone bang-sensitive flies sesB and heat-sensitive seiTS, there are no alterations to sleep pattern, but sleep deprivation leads to increased seizure severity. However, another study by Petruccelli et al., 2015 reported reduced seizure likelihood and increased seizure threshold after sleep deprivation in GEFS+ flies. Clearly, in fly models of epilepsy, like in human patients, the disease is heterogenous and each mutation potentially has a distinct relationship to sleep and its effect on susceptibility to seizure. This research combined with our data, which lacks a clear and consistent trend, suggests that sleep and seizure relationship could be investigated further for each genotype individually to better understand the underlying mechanisms. “

We cannot state if irregular activities reported lead to seizure susceptibility or seizure aggravation due to lack of investigation and the current data are not conclusive enough to determine the relationship, which has not been a focus of our study.

14. Figure 3: The finding of a consistent larval locomotion defect phenotype in bang-sensitive mutants is an important one. Does this affect recovery from larval electroshock?

We do not suggest there to be a clear effect between recovery from electroshock and locomotion. The below statement has been included in the discussion to address the comment:

"Moreover, there does not seem to be a clear relationship between locomotion and recovery time from electroshock in larvae. Bang-sensitive lines like pksple, jus and eas have reduced locomotion but their recovery time is comparable to DS and GEFS+ which display higher levels of locomotive activity.”

15. Figure 5: The authors don’t completely discuss their data set. What does CS EtOH mean?

To address this comment, we have updated Figure 5.

In Figure 4 we introduce PTX fed flies. PTX is dissolved in EtOH i.e. vehicle. We labeled this vehicle control as “-PTX” in Fig4, but we did not match the label in Fig5. “CS EtOH” and “CS -PTX” are the same group.

Discussion:

16. One of the general conclusions of the paper is that larval electroshock is a “broadly applicable [seizure] induction method” and is a recommended assay for hyperexcitable mutants. How does the approach compare to electroconvulsive seizure methods applied in adults? In adults the timing of distinct phases of the electroconvulsive seizure discharge seizure sequence is quite stereotypic (e.g. the delayed seizure discharge onset is +/- 3 s following a ∼20 s paralysis phase).

We addressed the comment by including the paragraph below in the discussion:

"Larval electroshock could be compared to adult electroshock. Adult electroshock successfully induces seizures in a range of bang-sensitive mutants including eas, bss and jus tested in our study (Pavlidis and Tanouye 1995; Pavlidis et al. 1994). There is a stereotypical sequence of seizure in adults including two distinct phases of dorsal longitudinal muscle failure defined as a silent period lasting 35 - 40 s and abnormal responses during recovery which varied by genotype. However, adult electroshock failed to induce seizures in temperature sensitive mutants like seizure. By contrast, our results indicate that electroshock of larvae successfully induced seizures in all mutants and, moreover, generated a comparable hierarchy of seizure severity to adults. Similarly to adult electroshock, the response is quite stereotyped in stages. It starts with larval paralysis of varied duration depending on genotype. This is followed by both or either partial peristaltic waves and mouth hook contractions of high frequency. Recovery is determined once several full peristaltic waves occur in the animal. “

Reviewer 2:

The manuscript nicely compares different seizure mutants with a large variety of assays to determine which assays reflect the underlying severity of the mutant. I believe that this careful work will accelerate the progress in the field by showing the utility of larval electroshock as a reliable method for assaying seizure mutants. Other work has looked at some of these methods but no paper has used so many methods and applied it to so many of these mutants.

We thank the Reviewer for their comments.

1. The writing could be more precise and more credit should be given to earlier studies on characterizing different seizure-prone mutants.

We have addressed this comment by updating some references and adding more information in the discussion as requested by Reviewer 1.

2. The authors should also address why they observe temperature sensitivity in the bss line while others have not seen this phenotype.

> We address this comment earlier as requested by Reviewer 1. We have updated Figure 2 to reflect the new set of experiments done.
